# Effects of Adding Posterior Ankle Joint Mobilization to Eccentric Training on Ankle Range of Motion and Athletic Performance in Basketball Athletes with Restricted Ankle Dorsiflexion: A Randomized Controlled Trial

**DOI:** 10.3390/jfmk11010092

**Published:** 2026-02-25

**Authors:** Vasileios Georgoulas, Ilias Kallistratos, Thomas Apostolou, Konstantinos Kasimis, Dimitrios Lytras, Paris Iakovidis

**Affiliations:** 1 Laboratory of Biomechanics & Ergonomics, Department of Physiotherapy, Faculty of Health Sciences, International Hellenic University—Alexander Campus, 57400 Sindos, Thessaloniki, Greece; vasilisgeorgoulas@gmail.com (V.G.); apostolouthomas@ihu.gr (T.A.); konstantinoskasimis@gmail.com (K.K.); piakov@ihu.gr (P.I.); 2 Laboratory of Basic and Applied Research in Physiotherapeutic Rehabilitation, Department of Physiotherapy, Faculty of Health Sciences, International Hellenic University, 57400 Sindos, Thessaloniki, Greece; elikall@ihu.gr

**Keywords:** ankle dorsiflexion, joint mobilization, basketball, eccentric exercise, athletic performance

## Abstract

**Background:** Restricted ankle dorsiflexion is common in basketball athletes and has been associated with altered lower-limb mechanics and reduced athletic performance. Although ankle joint mobilization is widely used to improve mobility, its effects on athletic performance remain unclear. The aim of this study was to examine whether adding posterior ankle joint mobilization to a structured exercise-based program incorporating eccentric strengthening and stretching improves ankle mobility and athletic performance in basketball athletes with restricted dorsiflexion. Primary outcomes were dorsiflexion range of motion (DF-ROM) and the Weight-Bearing Lunge Test (WBLT); secondary outcomes included jump performance, hop tests, Reactive Strength Index, Fatigue Index, and maximal isometric strength. **Methods:** In this randomized controlled trial, 38 basketball athletes (mean age 21.26 ± 2.52 years) with unilateral restricted ankle dorsiflexion were randomly allocated to an exercise-only group (*n* = 19) or to an exercise plus talocrural mobilization group (n = 19). The intervention lasted 5 weeks, with assessments performed at baseline, post-intervention, and at a 3-month follow-up. **Results:** Both groups improved ankle dorsiflexion; however, greater gains were observed in the intervention group for both dorsiflexion range of motion (DF-ROM; interaction *p* < 0.001; mean difference [MD] = 3.52° post-intervention and MD = 5.17° at follow-up) and the Weight-Bearing Lunge Test (WBLT; interaction *p* < 0.001; MD = 1.39 cm and MD = 1.34 cm, respectively). The intervention group showed superior improvements in countermovement jump and Triple Hop Test performance (both *p* < 0.001), as well as a small but statistically significant advantage in the Single Hop Test (*p* = 0.015). No between-group differences were found for the 6 m timed hop test, Reactive Strength Index, Fatigue Index, or maximal isometric strength (*p* > 0.05). **Conclusions:** Adding ankle joint mobilization to an eccentric strengthening and stretching program produced greater improvements in dorsiflexion and jump performance than exercise alone, without affecting speed, reactive ability, or maximal strength. Ankle mobilization may be a useful adjunct for improving functional mobility and selected performance outcomes in basketball athletes.

## 1. Introduction

Restricted ankle dorsiflexion range of motion (DF-ROM) has been consistently associated with altered landing mechanics and the adoption of stiffer and compensatory movement strategies, which modify load distribution and increase mechanical demands on the lower limb during landing tasks [1,2,3,4,5]. Adequate ankle dorsiflexion is considered a key determinant of efficient lower-limb function and athletic performance, particularly in sports characterized by frequent jumps, landings, and rapid changes in direction [6]. When ankle mobility is restricted, biomechanical adaptations emerge that alter landing mechanics, reduce movement efficiency, and increase mechanical stress along the kinetic chain [7].

More specifically, limited DF-ROM promotes “stiffer” landing strategies, characterized by reduced ankle–knee–hip excursions and higher loading rates, even in the absence of substantial changes in peak vertical ground reaction force [8,9,10]. These patterns are particularly relevant in basketball, a sport characterized by repeated jumps, landings, abrupt changes in direction, and pivots, which impose high and repetitive mechanical loads on the lower limbs and especially on the ankle joint [11]. Under such conditions, dorsiflexion restrictions influence not only joint-level mobility but also global movement strategies and neuromechanical loading profiles [7].

From a mechanistic perspective, reductions in ankle dorsiflexion may arise not only from soft-tissue shortening and reduced joint play, but also from alterations in joint axis alignment and talocrural arthrokinematics, highlighting the multifactorial nature of dorsiflexion limitations and the need to address both tissue-related and joint-related contributors [12].

In applied sports and clinical settings, the Weight-Bearing Lunge Test (WBLT or knee-to-wall, KTW) is widely accepted as a reliable measure of closed-chain ankle dorsiflexion [13,14,15]. Several intervention strategies have been proposed to improve DF-ROM. Stretching remains the most commonly used approach [16], while eccentric exercise can increase muscle length under load and contribute to flexibility adaptations [17]. In parallel, manual therapy techniques targeting joint mechanics have attracted substantial interest. In particular, posterior talar glide techniques aim to restore talocrural arthrokinematics and are widely used to improve ankle dorsiflexion [18], with reported benefits in joint mobility that are essential for activities such as running, jumping, and rapid changes in direction [6,19].

Mobilization-with-movement (MWM) interventions, especially those emphasizing posterior talar glide, directly target talocrural arthrokinematics and have been shown to induce immediate increases in DF-ROM [20,21]. At the same time, stretching and eccentric exercise programs have also been reported to improve DF-ROM [17,22]. However, despite consistent evidence that different approaches can increase ankle dorsiflexion, several studies have reported inconsistent associations between changes in range of motion and improvements in functional or athletic performance [22,23].

It should be noted that jump propulsion is primarily driven by plantarflexor function and stretch–shortening cycle efficiency, whereas ankle dorsiflexion mainly influences landing and braking mechanics and the transition to take-off. Therefore, improvements in dorsiflexion are expected to affect jump performance indirectly, rather than through a direct propulsive mechanism.

Importantly, it remains unclear whether the manner in which dorsiflexion is improved—through restoration of joint arthrokinematics versus increases in soft-tissue extensibility—determines the degree to which these gains transfer to functional and athletic performance outcomes. This uncertainty is also reflected in the conclusions of recent evidence syntheses. Mason-Mackay et al. [7] reported that restricted dorsiflexion is associated with altered landing biomechanics and movement strategies that increase lower-limb loading. Vallandingham et al. [24] showed that joint mobilization techniques can produce small-to-moderate improvements in DF-ROM, although effects on dynamic postural control were less consistent. More recently, Paula et al. [25] found a statistically significant benefit of manual therapy on weight-bearing dorsiflexion in sham-controlled trials, but emphasized that the overall certainty of evidence remains very low due to substantial heterogeneity and imprecision. Collectively, these findings support the biomechanical relevance of dorsiflexion and the capacity of different interventions to improve it, while leaving unresolved the critical question of performance transfer.

Therefore, the purpose of the present randomized controlled trial was to investigate whether an intervention targeting talocrural arthrokinematics through posterior talar mobilization-with-movement combined with eccentric exercise would produce greater and more functionally transferable improvements in ankle dorsiflexion and basketball-relevant performance outcomes than a program based on stretching and eccentric exercise alone, in young, asymptomatic basketball athletes with restricted ankle dorsiflexion.

## 2. Materials and Methods

### 2.1. Study Design

This study was designed as a parallel-group randomized controlled trial (RCT) and conducted in accordance with the CONSORT guidelines under the scientific supervision of the Department of Physiotherapy, School of Health Sciences, International Hellenic University. The study protocol was approved by the Research Ethics Committee of the Department of Physiotherapy (approval number: EC-02/2025) and registered in ClinicalTrials.gov prior to data collection (ID: NCT06828744). The trial followed a single-blind design, with outcome assessors blinded to group allocation; blinding of participants and treating physiotherapists was not feasible due to the nature of the intervention. Recruitment and data collection were carried out between March and July 2025 at the facilities of the International Hellenic University in Thessaloniki, Greece. The intervention period lasted five weeks, and assessments were performed at baseline, post-intervention (week 5), and at a 3-month follow-up. All participants provided written informed consent in accordance with the Declaration of Helsinki.

### 2.2. Participants

The study sample consisted of young basketball athletes training regularly who presented with a unilateral restriction in ankle dorsiflexion. Participants were recruited from the International Hellenic University and organized basketball training facilities in the wider Thessaloniki area through an open call and underwent eligibility screening.

Eligible participants were active basketball players of either sex, aged 18–25 years, with a unilateral restriction in ankle dorsiflexion documented by the Weight-Bearing Lunge Test (side-to-side difference ≥ 2 cm), who reported no ankle pain at the time of inclusion and provided written informed consent. Regular participation in sports training for at least one month prior to the intervention was also required.

Exclusion criteria included a history of lower-limb surgery, lower-limb musculoskeletal injury within the previous six months, neurological, vestibular, or balance disorders, diagnosed connective tissue or other systemic diseases affecting ankle function, and inability to comply with the intervention or assessment procedures.

### 2.3. Randomization and Blinding

After completion of the eligibility screening, participants were randomly allocated to either the intervention group or the control group using a computer-generated random sequence, with stratification by sex to ensure balanced group distribution. Group allocation was performed by an independent member of the research team who was not involved in outcome assessment or intervention delivery, thereby ensuring allocation concealment. The study used a single-blind design, with outcome assessors blinded to group allocation to minimize measurement bias. Due to the nature of the intervention, blinding of participants and care providers was not feasible.

### 2.4. Measurements

All outcome measures were assessed by an independent assessor blinded to group allocation. Primary outcomes were active ankle dorsiflexion range of motion (DF-ROM) and the Weight-Bearing Lunge Test (WBLT). Secondary outcomes included the countermovement jump (CMJ), hop tests (Single Hop, Triple Hop, and 6 m Timed Hop), Reactive Strength Index (RSI), Fatigue Index (FI), and maximal isometric ankle strength (DFMVC and PFMVC). All outcomes were measured at three time points: baseline, immediately after the intervention (week 5), and at the 3-month follow-up.

#### 2.4.1. Active Ankle Dorsiflexion Range of Motion (DF-ROM)

DF-ROM was measured in degrees using a digital goniometer (GemRed 12″ Digital Goniometer, Guilin GemRed Sensor Technology Co., Ltd., China) with participants seated and the knee slightly flexed (~20°). Three trials were recorded and averaged. Goniometric ankle ROM assessment is widely used, with reliability depending on strict standardization and examiner experience [26]. Previous studies report low-to-moderate intra- and inter-rater reliability for active dorsiflexion (ICC = 0.32–0.72), especially between examiners [27]; nevertheless, it is considered an acceptable method when standardized procedures are applied and shows acceptable agreement with more complex kinematic systems [28].

#### 2.4.2. Weight-Bearing Lunge Test (WBLT)

Weight-bearing ankle dorsiflexion was assessed using the WBLT, with participants standing facing a wall and progressively advancing the tibia while keeping the heel in contact with the ground; the toe-to-wall distance was measured in centimeters using a standard measuring tape. Three trials were recorded and averaged [29]. The WBLT is considered a functional measure of ankle dorsiflexion and more representative of sport-specific loading conditions than non–weight-bearing assessments [30]. The test demonstrates excellent intra- and inter-rater reliability, with ICC values > 0.95 [31] and ranging from 0.97 to 0.99 in athletic populations [30]. A systematic review reported good-to-excellent reliability (ICC 0.65–0.99 intra-rater and 0.80–0.99 inter-rater) and adequate sensitivity to change, with a minimal detectable change (MDC) of approximately 1.5–2.0 cm when measured in centimeters [29].

#### 2.4.3. Countermovement Jump (CMJ)

Explosive lower-limb power was assessed using the CMJ test, recorded with an optical measurement system (Optojump Next, Microgate, Bolzano, Italy), which calculates jump height from flight time. Participants performed one familiarization trial followed by three valid jumps with at least 60 s rest, and the mean value was used for analysis. The CMJ is a widely used and valid measure of lower-limb explosive power and stretch–shortening cycle function, showing high intra- and inter-rater reliability (ICC > 0.90) in athletic populations [32]. The Optojump system demonstrates high validity and reliability with very small measurement error for CMJ height when standardized protocols are applied [33].

#### 2.4.4. Hop Tests (Single Hop, Triple Hop, and 6 m Timed Hop)

Functional athletic performance was assessed using a battery of hop tests (Single-Leg Hop for Distance, Triple Hop for Distance, and 6 m Timed Hop), widely used to evaluate lower-limb explosive power, dynamic stability, and neuromuscular function [34,35]. Tests were performed in a standardized order after a warm-up, first on the non-tested and then on the tested limb. One familiarization trial and two valid trials were recorded for each test, and the mean value was used for analysis.

The Single Hop and Triple Hop measured the maximal horizontal distance (cm) using a standard measuring tape placed along the testing surface, whereas the 6 m Timed Hop recorded the time (s) required to cover a 6 m distance on one leg using a handheld digital stopwatch (Amila 44092 Professional Stopwatch, Amila Sports, Athens, Greece). Hop tests have demonstrated high reliability and validity for assessing functional performance and limb symmetry, with reported ICC values > 0.90 for both the Single and Triple Hop tests [35], and excellent reproducibility in athletic populations [34]. The Triple Hop has also shown strong associations with vertical jump height and isokinetic strength, supporting its validity as a marker of lower-limb power [36]. Time-based hop tests, including the 6 m Timed Hop, have also demonstrated high reliability and sensitivity for detecting functional asymmetries and performance deficits [35,37].

#### 2.4.5. Reactive Strength Index (RSI)

Reactive strength was assessed using the RSI, a measure of the ability to rapidly transition from eccentric to concentric muscle action during stretch–shortening cycle activities. Participants performed repeated jump contacts with minimal ground contact time, and RSI was calculated as jump height (m) divided by ground contact time (s), with the mean value of valid trials used for analysis. The RSI is commonly used as an indicator of reactive neuromuscular performance. It has demonstrated high reliability and sensitivity to performance changes in athletic populations and is considered a reliable marker of stretch–shortening cycle function when measured using standardized protocols and optical systems [33].

#### 2.4.6. Fatigue Index (FI)

Lower-limb fatigue resistance was assessed using a FI, calculated as the percentage decline in performance between the initial and final phases of repeated single-leg hop efforts. FI was computed as the relative change (%) between initial and final performance values, with higher values indicating greater performance decrement. The FI was used to complement absolute performance measures by capturing the ability to maintain functional output under repeated loading conditions, which is particularly relevant in high-intensity sports such as basketball. Analysis of performance changes during repeated functional hop tasks has been widely used to identify deficits not evident in single-effort tests [34] and to provide complementary information to absolute performance values [35]. Recent evidence also suggests that interpretation of hop test performance should consider not only symmetry but also performance maintenance across repetitions [37]. Accordingly, FI was used in the present study as an interpretative indicator of functional fatigue rather than as an independent psychometric test with established validity thresholds.

#### 2.4.7. Isometric Ankle Muscle Strength

Maximal isometric strength of the ankle dorsiflexors and plantar flexors was assessed using a handheld dynamometer (Model 01165, Lafayette Instrument Company, Lafayette, IN, USA) following a standardized protocol. Measurements were performed in a seated position with the hip and knee at approximately 90° and the ankle in neutral position. Three maximal 3–5 s contractions were recorded for each muscle group, and the highest value (N) was used for analysis. Handheld dynamometry has been shown to provide good to excellent reliability for lower-limb strength assessment when standardized positioning and stabilization are applied [38], with reported ICC values typically ranging from 0.80 to >0.95. Specifically for ankle plantar flexors, high test–retest reliability has been reported (ICC = 0.91–0.94), with an MDC corresponding to approximately 12–15% of the mean strength value under stabilized conditions [39]. Additional evidence indicates excellent intrarater reliability (ICC > 0.93) and low measurement error when stabilization is used [40]. These data support the use of handheld dynamometry as a reliable method for detecting clinically meaningful changes in ankle isometric strength.

### 2.5. Experimental Protocols

All participants continued their regular basketball team training throughout the study period, including technical, tactical, and plyometric drills, under the supervision of their team strength-and-conditioning coaches. Training loads were comparable between groups, and no additional lower-limb flexibility, strengthening, or manual therapy interventions were permitted during the study period. Both groups received identical session frequency, duration, exercise components, and supervision; only the manual therapy differed (posterior talar glide vs. static stretching).

#### 2.5.1. Exercise Only Group

Participants in the control group followed a standardized exercise program consisting of eccentric strengthening and stretching exercises targeting the ankle plantar flexors, aiming to improve ankle dorsiflexion and lower-limb function. The program was delivered in two supervised sessions per week for five weeks by a physiotherapist certified in Orthopaedic Manual Physical Therapy (OMPT), with each session lasting approximately 25 min. The eccentric strengthening component consisted of eccentric heel-lowering exercises emphasizing both the gastrocnemius (knee extended) and the soleus (knee slightly flexed). Exercises were performed on the edge of a step, starting from a plantarflexed position and lowering the heel below step level through the full available dorsiflexion range. The concentric phase was performed bilaterally, followed by unilateral eccentric lowering on the tested limb. Participants were allowed light hand support for balance. Each eccentric lowering phase was performed in a controlled manner over approximately 3–4 s. Participants performed three sets of 15 repetitions. In addition, step-lunge dorsiflexion drills were performed to promote functional dorsiflexion under load. The stretching component included static self-stretching exercises for the gastrocnemius and soleus muscles performed in a lunge position, with each stretch held for 30 s and repeated three times per session, at an intensity corresponding to mild discomfort. Exercise load and progression were adjusted individually by increasing the range of motion and controlled tempo as tolerated, while maintaining correct technique and absence of pain. Training volume and intensity were individually adjusted to ensure proper technique and the absence of pain. Rest intervals between sets were approximately 60 s. The total training volume was kept consistent across participants.

#### 2.5.2. Exercise Plus Talocrural Mobilization Group

Participants in the intervention group followed the same eccentric strengthening and stretching program as the control group and additionally received a manual therapy program targeting talocrural joint arthrokinematics. The supervised intervention sessions were delivered twice per week for five weeks by the same OMPT-certified physiotherapist and had a similar overall duration (~25 min). The manual therapy protocol included posterior talar glide mobilization with active dorsiflexion according to the Mulligan mobilization-with-movement (MWM) concept, applied for three sets of 10 repetitions using grade III–IV oscillatory mobilizations with an approximate duration of 2–3 s per repetition. These mobilizations were applied with the participant actively dorsiflexing the ankle while the therapist applied a posterior glide to the talus relative to the tibia, within a pain-free range of motion. In addition, posterior talar glide mobilizations were applied in non–weight-bearing conditions, as well as weight-bearing MWM using a stabilization belt placed around the distal tibia/ankle and anchored posteriorly, while the participant actively dorsiflexed the ankle within a pain-free range. All mobilization applications were considered components of the same MWM protocol, and each mobilization technique was applied for approximately three sets, with short rest intervals between sets. Furthermore, participants were instructed in a self-mobilization technique using a belt, performed in a weight-bearing lunge position, which was performed daily at home for three sets of 30–60 s.

### 2.6. Sample Size Estimation

The required sample size was estimated using G*Power (version 3.1.9.7) based on a two-way repeated-measures ANOVA with within–between interaction (the planned statistical model). The calculation was based on the primary outcomes (DF-ROM and WBLT). Assumptions were set at α = 0.05, power (1−β) = 0.90, two groups, three measurement time points, and an expected correlation among repeated measures of 0.50. A moderate effect size was assumed (Cohen’s f = 0.25) according to Cohen [41], and the nonsphericity correction was set to ε = 1. The analysis indicated a minimum total sample of 36 participants (actual power = 0.91); allowing for an additional 5% attrition, the target sample size was set at 38 participants.

### 2.7. Statistical Analysis

Statistical analyses were performed using SPSS (version 25.0; IBM Corp., Armonk, NY, USA). Descriptive statistics were computed for all variables. Normality of distribution was assessed using the Shapiro–Wilk test and Q–Q plots. As all variables were normally distributed, data are presented as means ± standard deviations. Baseline between-group differences in demographic and anthropometric characteristics were examined using independent-samples *t*-tests for continuous variables and chi-square tests for categorical variables.

To examine the effects of the intervention, a two-way repeated-measures ANOVA (group × time) was performed separately for each dependent variable. DF-ROM and WBLT were defined as primary outcomes, whereas all other variables were considered secondary outcomes. The between-subject factor was group (intervention vs. control), and the within-subject factor was time (baseline, week 5, and 3-month follow-up). Assumptions of homogeneity of variances (Levene’s test) and sphericity (Mauchly’s test) were checked, and when sphericity was violated, the Greenhouse–Geisser correction was applied. When significant main effects or interactions were found, post hoc comparisons were conducted using paired or independent *t*-tests, as appropriate, with Bonferroni adjustment for multiple comparisons.

Effect sizes were calculated using partial eta squared (η^2^p) for ANOVA effects to quantify the magnitude of group, time, and interaction effects. The level of statistical significance was set at *p* < 0.05.

## 3. Results

**Figure 1:**
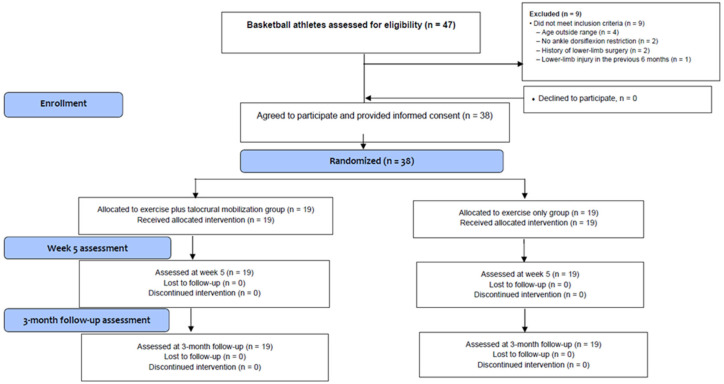
CONSORT Flow Diagram of the Study.

During the recruitment period (March 2025 to July 2025), a total of 47 basketball athletes were screened for eligibility. Of these, 38 participants met the predefined inclusion criteria and were enrolled in the study. Recruitment was completed once the required sample size, as determined by the a priori sample size calculation, was achieved. The flow of participants through the stages of screening, randomization, and intervention is presented in the CONSORT flow diagram (Figure 1). No participants were lost to follow-up after enrollment, and no deviations from the study protocol were recorded.

No adverse events, complications, or intervention-related symptoms were reported during the study period. All participants completed the intervention and all scheduled assessments according to the predefined protocol.

Detailed demographic and athletic characteristics of the participants in each group, along with between-group comparisons, are presented in Table 1.

### 3.1. Ankle Dorsiflexion Range of Motion (DF-ROM)

A significant group × time interaction was observed for DF-ROM (*p* < 0.001), indicating a differential response between groups over time. Both groups showed significant improvements in ankle dorsiflexion from baseline to post-intervention and follow-up (*p* < 0.001) (Table 2). However, the exercise plus mobilization group demonstrated significantly greater gains compared with the exercise-only group. At post-intervention, the intervention group exhibited higher DF-ROM values than the control group (mean difference = 3.52°, 95% CI: 2.37–4.66, *p* < 0.001), and this between-group difference further increased at the 3-month follow-up (mean difference = 5.17°, 95% CI: 4.24–6.11, *p* < 0.001). The between-group difference in DF-ROM was statistically significant at both post-intervention and the 3-month follow-up.

### 3.2. Weight-Bearing Lunge Test (WBLT)

A significant group × time interaction was found for WBLT (*p* < 0.001), indicating a differential response between groups over time. Both groups showed significant improvements from baseline to post-intervention and follow-up (*p* < 0.001) (Table 2). However, the exercise plus mobilization group demonstrated significantly greater improvements compared with the exercise-only group. At post-intervention, the intervention group achieved higher WBLT values than the control group (mean difference = 1.39 cm, 95% CI: 1.12–1.65, *p* < 0.001). This between-group difference remained significant at the 3-month follow-up (mean difference = 1.34 cm, 95% CI: 1.12–1.56, *p* < 0.001).

### 3.3. Single Hop Test (SHT)

A significant group × time interaction was observed for SHT (*p* = 0.015), indicating different performance trajectories between groups. The exercise plus mobilization group showed significant improvements from baseline to post-intervention and follow-up (*p* < 0.001), whereas no significant changes were observed in the exercise-only group. Between-group comparisons revealed no difference at baseline (*p* = 0.907) (Table 2). However, the intervention group demonstrated significantly better performance than the control group at post-intervention (*p* = 0.006) and at the 3-month follow-up (*p* = 0.007).

### 3.4. Triple Hop Test (THT)

A significant group × time interaction was observed for THT (*p* < 0.001), indicating different performance trajectories between groups. Both groups showed significant improvements over time; however, the exercise plus mobilization group demonstrated greater gains than the exercise-only group (Table 2). Between-group comparisons revealed no difference at baseline (*p* = 0.293). At post-intervention, the intervention group achieved higher THT values than the control group (mean difference = 7.20 cm, 95% CI: 4.89–9.51, *p* < 0.001), and the between-group difference was larger at the 3-month follow-up (mean difference = 12.89 cm, 95% CI: 9.47–16.31, *p* < 0.001).

### 3.5. Single-Leg 6 m Timed Hop Test (SLHT-6M)

A significant main effect of time was observed for SLHT-6M (*p* < 0.001), indicating overall improvement in performance (i.e., reduced completion time) in both groups (Table 2). However, no significant group × time interaction was found (*p* = 0.198), indicating that the magnitude of improvement did not differ between the exercise plus mobilization group and the exercise-only group. Both groups demonstrated similar reductions in completion time from baseline to post-intervention and follow-up.

### 3.6. Countermovement Jump (CMJ)

A significant group × time interaction was observed for CMJ (*p* < 0.001), indicating different performance trajectories between groups (Table 2). Both groups showed improvements over time; however, the exercise plus mobilization group demonstrated significantly greater gains compared with the exercise-only group. Between-group comparisons revealed no difference at baseline (*p* = 0.241). At post-intervention, the intervention group achieved higher jump height than the control group (mean difference = 1.17 cm, 95% CI: 0.79–1.54, *p* < 0.001), and this difference further increased at the 3-month follow-up (mean difference = 1.80 cm, 95% CI: 1.36–2.24, *p* < 0.001).

### 3.7. Reactive Strength Index (RSI)

A significant main effect of time was observed for RSI (*p* < 0.001), indicating overall improvement in reactive strength in both groups (Table 2). However, no significant group × time interaction was found (*p* = 0.453), indicating that the magnitude of improvement did not differ between the exercise plus mobilization group and the exercise-only group. Both groups showed similar increases in RSI from baseline to post-intervention and follow-up.

### 3.8. Fatigue Index (FI)

A significant main effect of time was observed for FI (*p* < 0.001), indicating an overall reduction in fatigue in both groups over time. However, no significant group × time interaction was found (*p* = 0.438), indicating that the magnitude of improvement did not differ between the exercise plus mobilization group and the exercise-only group (Table 2). Both groups demonstrated similar reductions in FI from baseline to post-intervention and follow-up.

### 3.9. Maximal Isometric Dorsiflexor Strength (DFMVC)

No significant main effect of time was observed for DFMVC (*p* = 0.154), and no significant group × time interaction was found (*p* = 0.735) (Table 2). Maximal isometric dorsiflexor strength did not change significantly over time in either group, and no between-group differences were observed.

### 3.10. Maximal Isometric Plantarflexor Strength (PFMVC)

A significant main effect of time was observed for PFMVC (*p* < 0.001), indicating a small overall change in plantarflexor strength over time in both groups (Table 2). However, no significant group × time interaction was found (*p* = 0.276), indicating that the magnitude of change did not differ between the exercise plus mobilization group and the exercise-only group.

## 4. Discussion

The present study investigated the effects of adding ankle joint mobilization to a structured eccentric strengthening and stretching program in basketball athletes with restricted ankle dorsiflexion, both in terms of joint mobility and selected performance outcomes. Overall, the findings indicate that this addition not only produced greater improvements in ankle dorsiflexion, but that these improvements were also partly transferred to specific indicators of athletic performance. Indeed, improvements were observed not only in ankle mobility (ROM, WBLT), but also in several performance measures, including the SHT, THT, and CMJ. Notably, these benefits were already evident immediately after the intervention and became more pronounced at follow-up.

However, the positive effects on performance were not uniform across all examined outcomes, as several variables did not differ between groups. This finding likely reflects the fact that improvements in talocrural arthrokinematics cannot be directly or linearly translated into performance outcomes, which depend on complex, multijoint neuromuscular coordination and integrated movement patterns.

This interpretation is consistent with the view that the way ankle mobility is restored—through mechanisms primarily targeting talocrural arthrokinematics rather than merely changes in soft tissue extensibility—may influence both the magnitude and the temporal dynamics with which improvements are integrated into functional and sport-specific movement patterns [20,21,42,43]. Moreover, the fact that some explosive performance indices showed further improvement at the 3-month follow-up, compared with the immediate post-intervention assessment, likely reflects the time required for these neuromuscular adaptations to consolidate: initial gains in mobility and ankle mechanical behavior appear to be progressively incorporated into complex movement patterns through continued training exposure and neuromuscular adaptation [44,45]. From this perspective, the interpretation of the findings is not limited to statistical significance alone, but also considers whether the observed changes are likely to exceed measurement error and the corresponding MDC thresholds, where these have been established for ankle mobility and functional strength assessments [29,39].

Regarding the effects of the intervention on ankle dorsiflexion ROM, the findings indicate that the addition of ankle joint mobilization improved ROM both in the short and long term, under both open-chain conditions (as assessed by goniometric measurement of active ROM) and, more importantly, under closed-chain conditions as assessed by the WBLT. Although goniometry is a widely used clinical tool, its intra- and inter-rater reliability has been reported to be variable and, in several contexts, only moderate, particularly when different examiners are involved [26,27,28], which makes temporally consistent and between-group differentiated changes more clinically interpretable. In contrast, the WBLT exhibits high reliability and low measurement error [30,31,46] and has been shown to be sensitive to detecting changes exceeding the minimal detectable threshold [29]. This strengthens the interpretation that the larger and sustained increases observed in the intervention group reflect meaningful improvements in functional ankle mobility rather than random variation. Given that the WBLT approximates loading patterns relevant to athletic tasks such as landings and change-of-direction maneuvers [30], these findings suggest that the intervention improved not only the available range of motion, but also its functional expression under conditions of real mechanical demand.

Given that the MDC of the WBLT has been estimated to be approximately 1.5–2.0 cm [29], the overall improvement observed in the intervention group (>3 cm) clearly exceeds measurement error and can therefore be considered a real and reliable change in functional dorsiflexion. In contrast, the improvement in the control group remained close to the limits of the MDC, rendering its interpretation more cautious. Although the MDC is not directly applicable to between-group comparisons, the temporal stability of the superiority of the intervention group, together with the concurrent improvements observed in functional hopping performance, further supports the interpretation that the observed difference carries meaningful functional significance.

The observed between-group differences, both in the magnitude and in the temporal persistence of the improvements, may be interpreted in light of the different mechanisms through which ankle dorsiflexion is restored. Mobilization with movement has been described as an intervention that is hypothesized to directly target ankle arthrokinematics, particularly by facilitating posterior talar glide during dorsiflexion, thereby potentially improving joint surface congruency and reducing functional mechanical constraints to motion [42,43]. In contrast to interventions that primarily aim to increase soft tissue extensibility, joint mobilization may primarily influence the mechanical behavior of the joint itself, which could create conditions under which the available ROM can be used more effectively under load.

Beyond its purely mechanical effects, restoration of arthrokinematics has also been proposed to be accompanied by changes in sensorimotor control and proprioceptive input, which may facilitate the functional utilization of the increased range of motion within dynamic and multi-joint movement patterns [20,21,42]. From this perspective, joint mobilization does not act as a simple “ROM enhancer,” but rather as an intervention that modifies the mechanical and neuromuscular context within which range of motion is integrated into functional activities. This distinction provides a plausible explanation for why the improvements observed in the intervention group were not only greater, but also more stable over time and more readily transferable to demanding jump-based tasks.

The CMJ is a classic indicator of lower-limb explosive power and reflects the ability to exploit the stretch–shortening cycle through coordinated multi-joint motion of the hip, knee, and ankle [32,33]. In the present study, the intervention group exhibited significantly greater improvements in jump height both at post-intervention and at follow-up, with the between-group difference being more pronounced at the 3-month follow-up. This temporal pattern suggests that changes in ankle mobility may be progressively integrated into jumping performance over time [47,48].

One possible explanation is that increased functional ankle dorsiflexion allows a larger and more controlled range of motion during the braking phase, thereby enhancing load absorption capacity and elastic energy storage prior to the subsequent propulsive phase [47]. The additional improvement observed at the 3-month follow-up may reflect the time required for neuromuscular adaptation to these mechanical advantages, which is achieved primarily through repeated exposure to jumping and sport-specific activities until these more efficient movement strategies become established. This interpretation is consistent with previous reports indicating that improvements in complex expressions of power often emerge with a delay relative to the initial changes in mobility or joint mechanical function [44,45].

Regarding the hop tests, the Triple Hop for Distance demonstrated the clearest and most clinically meaningful between-group differentiation, with the intervention group showing significantly greater improvements, which were further amplified at the 3-month follow-up. This finding is not unexpected, given that the Triple Hop is not merely a measure of maximal single-leg propulsion, but rather a complex task involving repeated loading, force absorption, and re-propulsion, which requires stable and functionally integrated ankle mobility across successive stretch–shortening cycles [34,36,47]. From this perspective, the superior performance of the intervention group is consistent with the notion that improved functional dorsiflexion does not simply act as a mechanical advantage in a single jump, but facilitates the maintenance of a more efficient movement strategy under conditions of cumulative mechanical demand.

In contrast, the Single-Hop Test, although it revealed a statistically significant group × time interaction and a superiority of the intervention group at both post-intervention and follow-up, exhibited a clearly smaller magnitude of change. This finding can be interpreted in light of the lower motor complexity of this task, as a single maximal-effort jump allows compensation for ankle-related constraints through hip and knee strategies and, therefore, shows reduced sensitivity for detecting differentiated effects arising from mobility-focused interventions [37]. Moreover, in athletes with a high baseline level of performance, a ceiling effect is likely to occur, further limiting the ability to detect large between-group differences even when genuine functional improvement is present.

Finally, the SLHT-6M did not show any between-group differentiation, despite a general improvement in both groups, suggesting that this outcome is dominated to a greater extent by execution speed and inter-step coordination and is therefore less sensitive to detect differentiated effects arising from ankle mobility–focused interventions, as has been previously suggested for timed hop performance tests in athletic populations [37]. Under this perspective, the observed improvements in the timed hop are more likely to reflect general training-induced adaptations during the competitive season rather than a specific effect of joint mobilization. Overall, the differential response pattern across the hop tests reinforces the notion that more complex and “cumulative” tasks are also more sensitive for detecting the functional integration of improved mobility.

Regarding the remaining performance outcomes, the present study showed that both the RSI and the FI improved significantly over time in both groups, without, however, a significant group × time interaction. This finding indicates that both groups demonstrated similar time-dependent improvements, without additional benefit attributable to the addition of joint mobilization.

The absence of a differential effect on these indices can be explained by their underlying nature. Both RSI and the FI primarily reflect parameters related to the speed and efficiency of the stretch–shortening cycle, neuromuscular activation, and the ability to sustain performance under repeated loading. Variables of this type are known to be influenced to a much greater extent by the overall training load, as well as by training-related stimuli common to both groups, and to a lesser degree by interventions that primarily target the mechanical restoration of joint mobility [47]. Given that no significant group × time interaction was observed, these factors should be interpreted as potential contributors rather than specific effects of joint mobilization.

From this perspective, the findings support the notion that ankle joint mobilization does not act as a direct or specific modulator of reactive muscle function or fatigue resistance. Instead, these indices appear to improve primarily as a consequence of the overall training process to which both groups were exposed. This observation is fully consistent with the broader pattern of the study’s results, according to which the addition of joint mobilization selectively affects performance variables that depend more strongly on the functional utilization of mobility and on movement mechanics (such as CMJ and Triple Hop), but not variables that primarily reflect the speed of neuromuscular activation and metabolic or neuromuscular endurance.

This differentiation reinforces the central interpretative conclusion of the present study: that ankle joint mobilization does not constitute a general “performance enhancer,” but rather exerts selective effects on those aspects of performance that depend on movement mechanical quality, the capacity for force absorption and reutilization, and the functional integration of mobility within multi-joint movement patterns.

The present findings showed that DFMVC did not exhibit a statistically significant change over time and did not differ between groups, whereas PFMVC showed a small main effect of time but no significant group × time interaction. This pattern indicates that the addition of ankle joint mobilization did not meaningfully affect maximal isometric force production capacity compared with the exercise-based program alone.

This finding is not unexpected given the nature of the intervention. Ankle joint mobilization primarily aims to improve the mechanical behavior of the joint and the available range of motion, rather than to induce hypertrophic or neuromuscular adaptations associated with maximal force production. Similarly, the applied exercise-based program was designed mainly to enhance explosive performance and stretch–shortening cycle function, rather than to systematically increase maximal isometric strength, which would require different, more specific training stimuli of high resistance and sufficient volume.

Moreover, the literature indicates that changes in maximal isometric strength that exceed the measurement error of handheld dynamometers usually require increases on the order of 12–15% or greater [39]. From this perspective, the small changes observed in both groups, although in some cases statistically detectable as a main effect of time (particularly for the plantarflexors), cannot be interpreted with confidence as clinically meaningful or functionally decisive changes in maximal strength.

More importantly, however, the absence of meaningful changes in maximal isometric strength does not negate the positive effects observed in indices of explosive and functional performance. On the contrary, it reinforces the interpretation that the improvements in the CMJ and the hop tests did not stem from an increase in absolute muscle strength, but rather from improvements in the mechanical quality of movement, the ability to absorb and reutilize force, and the functional use of the available ankle dorsiflexion range of motion.

This pattern is consistent with the contemporary view that performance in complex athletic skills does not depend solely on “how much” force can be produced, but on “how” this force is transmitted, directed, and utilized within high-speed, high-demand multi-joint movement patterns [47].

One of the most interesting and clinically meaningful findings of the present study concerns the temporal evolution of the observed adaptations after the end of the intervention. Although the largest improvements in functional ankle mobility were already evident at the end of the 5-week intervention, between-group differences in performance outcomes were clearer and, in some cases, more pronounced at the 3-month follow-up. This pattern was observed primarily in the THT and the CMJ, where the superiority of the intervention group was not only maintained but further amplified over time.

This delayed expression of functional and performance-related gains is consistent with the notion that adaptations in joint mechanical behavior and available mobility precede their full integration into complex, high-demand multi-joint movement patterns. In other words, increases in available range of motion and improvements in ankle arthrokinematics do not automatically translate into immediate performance gains, but rather require a period during which the neuromuscular system “learns” to exploit these new mechanical capacities within the real context of training and competition.

The fact that both groups continued to follow the same training and competitive schedule during the follow-up period lends particular interpretive value to the follow-up findings. Under these conditions, the progressive amplification of the intervention group’s superiority cannot be attributed to differences in training load, but rather suggests that the initial intervention established a different mechanical and functional “substrate” that was gradually expressed through continued exposure to repeated sport-specific demands.

This pattern is consistent with previous reports indicating that improvements in complex motor skills and jumping performance often emerge with a delay relative to the initial changes in mobility or muscle function, particularly in athletic populations who continue to train and compete concurrently [44,45]. In this context, the three-month period appears to have represented a critical time window for the emergence of the functional integration of the initial mechanical adaptations.

In variables in which large-magnitude changes clearly exceeded measurement error and were accompanied by consistent temporal patterns and between-group differences—such as the WBLT, the THT, and the CMJ—the interpretation in favor of the presence of meaningful functional adaptations is substantially stronger and methodologically better supported.

By contrast, caution is also warranted when interpreting changes in measures of muscle strength and explosive performance. For example, in maximal isometric strength, the literature suggests that changes on the order of 12–15% are typically required to exceed measurement error and be considered real [39]. From this perspective, the small-magnitude changes observed in some outcomes, despite being statistically detectable, cannot be interpreted with confidence as clinically meaningful or functionally decisive.

The findings of the present study have clear and readily applicable practical implications for the design of rehabilitation and injury-prevention programs in basketball players with documented restrictions in ankle dorsiflexion. The addition of joint mobilization to an existing exercise-based program does not appear to function as a stand-alone stimulus for immediate performance enhancement, but rather as a complementary factor that facilitates the functional utilization of mobility over time. From this perspective, the application of joint mobilization should not be viewed as a rapid “interventional fix” to improve performance, but as part of an integrated strategy for movement restoration and re-education. The results suggest that the primary contribution of the intervention lies in improving the mechanical quality of movement and in facilitating the integration of the available dorsiflexion range into complex motor patterns, which can subsequently be exploited more effectively through training.

From a practical standpoint, the data support the notion that the assessment of ankle mobility should be based primarily on functional, load-bearing measurement tools, such as the WBLT, rather than exclusively on open-chain measures. The use of such tools allows the detection of changes that are more closely related to the sport’s kinematic demands and reduces the risk of overinterpreting small or functionally trivial changes.

At the same time, the absence of differential effects on indices such as maximal isometric strength, the reactive strength index, and the fatigue index underscores that interventions primarily targeting mobility are not expected to induce uniform improvements across all performance domains. Accordingly, the integration of joint mobilization should be implemented as a complementary component rather than at the expense of specific strength, power, and reactive training stimuli, which require different and more targeted programming.

Finally, the present findings highlight the importance of long-term follow-up when evaluating interventions in athletic populations. The delayed expression of certain functional and performance adaptations suggests that clinical and training decisions should not be based solely on immediate post-intervention outcomes, but should also take into account the progressive and context-dependent integration of these changes within the actual sporting environment.

Despite the methodological rigor of the randomized controlled design, several limitations should be considered when interpreting the findings of the present study. First, the sample size, although sufficient to detect statistically significant differences in key mobility and functional performance variables, may have limited the ability to identify smaller or more specific effects in more complex outcomes, such as reactive strength or fatigue resistance.

Moreover, the inclusion of basketball players with a documented restriction in ankle dorsiflexion strengthens the internal validity of the study but simultaneously limits the generalizability of the findings to other athletic populations or to individuals without a clear mobility deficit. Future studies should examine the effects of similar interventions in athletes from different sports, performance levels, and mobility profiles.

Another limitation relates to the nature of the intervention and the inability to fully control all external training and competition-related factors during the follow-up period. Although both groups followed the same training program, individual differences in participation, training load, and match exposure may have influenced the temporal expression of certain adaptations. Nevertheless, this condition largely reflects the real-world sporting environment and enhances the ecological validity of the findings.

In addition, the study did not include direct kinematic or kinetic assessments (e.g., three-dimensional motion analysis or ground reaction force measurements), which limits the ability to more precisely elucidate the mechanisms through which improvements in ankle mobility translate into changes in functional performance. Future research incorporating such methodologies would allow a more detailed characterization of the underlying biomechanical and neuromuscular adaptations.

Finally, the interpretation of the results relied on the combined consideration of statistical significance and the psychometric properties of the assessment tools. Despite efforts to avoid overinterpretation, some thresholds of clinical meaningfulness remain debated in the literature, particularly for outcomes such as explosive power and reactive performance. Future studies with larger samples, different intervention protocols, graded mobilization dosages, and longer follow-up periods may help to further clarify both the mechanisms and the long-term functional consequences of restoring ankle dorsiflexion mobility.

## 5. Conclusions

This randomized controlled trial demonstrates that adding ankle joint mobilization to an exercise-based program in basketball players with restricted dorsiflexion results in a significantly greater and more durable improvement in ankle mobility, both in open-chain measures (goniometric assessment) and, more importantly, under functional weight-bearing conditions as assessed by the WBLT. The WBLT increased by an amount greater than expected from measurement variability alone, indicating a reliable improvement in functional dorsiflexion.

In parallel, the findings indicate that restoring mobility through joint mobilization can be selectively transferred to performance outcomes with higher mechanical and coordinative demands, such as the CMJ and the THT, and—to a lesser extent—the SHT, with a more pronounced between-group differentiation at the 3-month follow-up. In contrast, outcomes primarily reflecting execution speed, reactive performance, fatigue, and maximal isometric strength did not differ between groups, supporting the view that joint mobilization does not act as a global “performance enhancer,” but rather as a complementary intervention that facilitates the functional use of mobility within complex, multi-joint movement patterns.

Overall, these findings support the clinical and training value of integrating ankle joint mobilization into exercise-based programs when a documented dorsiflexion restriction is present, with the aim not only of increasing available range of motion but also of promoting its gradual functional integration into demanding sport-specific skills. In addition, the results highlight the importance of using functional, weight-bearing assessment tools and incorporating a follow-up period in order to capture the time-dependent transfer of mechanical adaptations to athletic performance.

## Figures and Tables

**Table 1:** Demographic and sport-related characteristics of the participants by group. Continuous variables are presented as mean ± standard deviation (SD) and categorical variables as number (n) and percentage (%).

Demographic and Sport Characteristics	Exercise Plus Talocrural Mobilization Group	Exercise Only Group	*p*-Value
Age (years)	21.05 ± 2.80	21.47 ± 2.22	0.61 ^a^
Sex (male/female)	73.7% (n = 14) Male 26.3% (n = 5) Female	73.7% (n = 14) Male 26.3% (n = 5) Female	1.00 ^b^
Height (cm)	185.42 ± 4.58	187.24 ± 7.18	0.35 ^a^
Body mass (kg)	80.86 ± 9.09	85.13 ± 11.70	0.21 ^a^
Body Mass Index (kg/m^2^)	23.45 ± 2.14	24.11 ± 1.71	0.30 ^a^
Training age (months)	55.89 ± 16.86	57.16 ± 16.56	0.81 ^a^
Playing position	Guard: 42.1% (n = 8) Forward: 36.8% (n = 7) Center: 21.1% (n = 4)	Guard: 47.4% (n = 9) Forward: 36.8% (n = 7) Center: 15.8% (n = 3)	0.90 ^b^
Limb dominance	Right: 84.2% (n = 16) Left: 15.8% (n = 3)	Right: 78.9% (n = 15) Left: 21.1% (n = 4)	0.67 ^b^
Affected lower limb	Right: 78.9% (n = 15) Left: 21.1% (n = 4)	Right: 73.7% (n = 14) Left: 26.3% (n = 5)	0.70 ^b^

^a^ Independent samples *t*-test. ^b^ Chi-square (χ^2^) test.

**Table 2:** Effects of the interventions on ankle mobility, functional performance, and strength outcomes.

Group	Baseline (Mean ± SD) *	Week 5 (Mean ± SD) *	Follow-Up (Mean ± SD) *	Mean Difference †	95% CI †	Time Effect F(p, η^2^p)	Interaction *p*-Value
DF-ROM (degrees)							
Exercise + talocrural mobilization	28.26 ± 1.13	35.75 ± 1.54	38.08 ± 1.23	+7.49	(6.57, 8.41)	F(2,72) = 247.25, *p* < 0.001, η^2^p = 0.873	<0.001
Exercise only	27.54 ± 1.21	32.24 ± 1.92	32.91 ± 1.58	+4.69	(3.46, 5.93)		
WBLT (cm)							
Exercise + talocrural mobilization	9.04 ± 0.37	11.82 ± 0.36	12.08 ± 0.26	+2.78	(2.53, 3.03)	F(1.04,37.51) = 986.66, *p* < 0.001, η^2^p = 0.965	<0.001
Exercise only	8.89 ± 0.17	10.43 ± 0.43	10.74 ± 0.39	+1.54	(1.33, 1.75)		
CMJ (cm)							
Exercise + talocrural mobilization	31.72 ± 0.65	32.90 ± 0.65	34.15 ± 0.64	+1.18	(0.72, 1.63)	F(2.72) = 58.74, *p* < 0.001, η^2^p = 0.620	0.001
Exercise only	31.46 ± 0.71	31.73 ± 0.48	32.35 ± 0.70	+0.27	(−0.16, 0.71)		
THT (cm)							
Exercise + talocrural mobilization	529.0 ± 2.5	549.0 ± 3.1	558.0 ± 6.3	+19.96	(18.26, 21.67)	F(2.72) = 390.78, *p* < 0.001, η^2^p = 0.916	<0.001
Exercise only	528.1 ± 3.1	541.8 ± 3.9	545.2 ± 3.8	+13.74	(11.43, 16.05)		
SHT (cm)							
Exercise + talocrural mobilization	186.42 ± 1.13	188.82 ± 1.38	189.01 ± 1.89	+2.41	(1.43, 3.38)	F(1.857) = 11.98, *p* < 0.001, η^2^p = 0.250	0.013
Exercise only	186.37 ± 1.08	186.87 ± 2.49	187.04 ± 2.28	+0.49	(−0.81, 1.80)		
SLHT-6m (s)							
Exercise + talocrural mobilization	2.64 ± 0.02	2.60 ± 0.02	2.58 ± 0.02	−0.04	(−0.05, −0.03)	F(2.72) = 82.37, *p* < 0.001, η^2^p = 0.696	0.198
Exercise only	2.63 ± 0.02	2.59 ± 0.02	2.58 ± 0.02	−0.03	(−0.05, −0.02)		
RSI							
Exercise + talocrural mobilization	0.852 ± 0.024	0.864 ± 0.030	0.877 ± 0.023	+0.011	(0.005, 0.018)	F(1.34,48.22) = 60.12, *p* < 0.001, η^2^p = 0.625	0.453
Exercise only	0.838 ± 0.025	0.846 ± 0.025	0.858 ± 0.023	+0.008	(0.002, 0.014)		
FI							
Exercise + talocrural mobilization	1.0416 ± 0.0102	1.0142 ± 0.0164	0.9968 ± 0.0160	−0.0274	(−0.0336, −0.0212)	F(1.15,41.52) = 142.17, *p* < 0.001, η^2^p = 0.798	0.438
Exercise only	1.0432 ± 0.0120	1.0189 ± 0.0152	1.0042 ± 0.0158	−0.0242	(−0.0354, −0.0130)		
DFMVC (Nm)							
Exercise + talocrural mobilization	38.98 ± 4.34	40.81 ± 5.19	39.71 ± 4.27	+1.83	(1.20, 2.45)	F(1.06,38.25) = 2.10, *p* = 0.154, η^2^p = 0.055	0.735
Exercise only	39.65 ± 3.66	41.07 ± 3.80	40.78 ± 3.78	+1.42	(0.82, 2.02)		
PFMVC (Nm)							
Exercise + talocrural mobilization	123.68 ± 9.92	124.32 ± 10.00	124.63 ± 9.76	+0.63	(−0.93, 0.33)	F(1.62,58.33) = 15.75, *p* < 0.001, η^2^p = 0.304	0.276
Exercise only	124.61 ± 9.50	125.36 ± 9.56	125.20 ± 9.79	+0.75	(−1.13, −0.38)		

* Values are presented as mean ± SD. † Mean Difference and 95% CI refer to within-group changes from baseline to Week 5.

## Data Availability

The datasets generated and analyzed during the current study are available from the corresponding author upon reasonable request.
